# HIV Infection Functionally Impairs Mycobacterium tuberculosis-Specific CD4 and CD8 T-Cell Responses

**DOI:** 10.1128/JVI.01728-18

**Published:** 2019-02-19

**Authors:** Patrizia Amelio, Damien Portevin, Jerry Hella, Klaus Reither, Lujeko Kamwela, Omar Lweno, Anneth Tumbo, Linda Geoffrey, Khalid Ohmiti, Song Ding, Giuseppe Pantaleo, Claudia Daubenberger, Matthieu Perreau

**Affiliations:** a Service of Immunology and Allergy, Lausanne University Hospital, University of Lausanne, Lausanne, Switzerland; b Swiss Tropical and Public Health Institute, Basel, Switzerland; c University of Basel, Basel, Switzerland; d Ifakara Health Institute, Bagamoyo, Tanzania; e EuroVacc Foundation, Lausanne, Switzerland; f Swiss Vaccine Research Institute, Lausanne University Hospital, University of Lausanne, Lausanne, Switzerland; Emory University

**Keywords:** CD4 T cells, HIV, *Mycobacterium tuberculosis*, exhaustion

## Abstract

Human immunodeficiency virus (HIV) infection is the major risk factor predisposing for Mycobacterium tuberculosis progression from latent tuberculosis infection (LTBI) to tuberculosis disease (TB). Since long-term-treated aviremic HIV-infected individuals remained at higher risk of developing TB than HIV-uninfected individuals, we hypothesized that progression from LTBI to pulmonary TB (PTB) might be due not only to CD4 T-cell depletion but also to M. tuberculosis-specific CD4 T-cell functional impairment. To test this hypothesis, M. tuberculosis-specific T-cell frequencies and cytokine profiles were investigated in untreated Tanzanian individuals suffering from LTBI (*n* = 20) or PTB (*n* = 67) and compared to those of untreated M. tuberculosis/HIV-coinfected individuals suffering from LTBI (*n* = 15) or PTB (*n* = 10). We showed that HIV infection significantly reduced the proportion of Th2 (interleukin 4 [IL-4]/IL-5/IL-13) producing M. tuberculosis-specific CD4 T cells and IL-2-producing M. tuberculosis-specific CD4 and CD8 T cells in individuals with LTBI or PTB (*P < *0.05). Interestingly, the loss of IL-2 production was associated with a significant increase of PD-1 expression on M. tuberculosis-specific CD4 and CD8 T cells (*P < *0.05), while the loss of Th2 cytokine production was associated with a significant reduction of Gata-3 expression in memory CD4 T cells (*P < *0.05). Finally, we showed that the serum levels of IL-1α, IL-6, C-reactive protein (CRP), IL-23, and IP-10 were significantly reduced in M. tuberculosis/HIV-coinfected individuals with PTB compared to those in HIV-negative individuals with PTB (*P < *0.05), suggesting that HIV infection significantly suppresses M. tuberculosis-induced systemic proinflammatory cytokine responses. Taken together, this study suggests that in addition to depleting M. tuberculosis-specific CD4 T cells, HIV infection significantly impairs functionally favorable M. tuberculosis-specific CD4 T-cell responses in Tanzanian individuals with LTBI or PTB.

**IMPORTANCE** Mycobacterium tuberculosis and human immunodeficiency virus (HIV) infections are coendemic in several regions of the world, and M. tuberculosis/HIV-coinfected individuals are more susceptible to progression to tuberculosis disease. We therefore hypothesized that HIV infection would potentially impair M. tuberculosis-specific protective immunity in individuals suffering from latent tuberculosis infection (LTBI) or active pulmonary tuberculosis (PTB). In this study, we demonstrated that M. tuberculosis/HIV-coinfected individuals have fewer circulating M. tuberculosis-specific CD4 T cells and that those that remained were functionally impaired in both LTBI and PTB settings. In addition, we showed that HIV infection significantly interferes with M. tuberculosis-induced systemic proinflammatory cytokine/chemokine responses. Taken together, these data suggest that HIV infection impairs functionally favorable M. tuberculosis-specific immunity.

## Abstract (precis)

Mycobacterium tuberculosis and human immunodeficiency virus (HIV) infections are coendemic in several regions of the world, and M. tuberculosis/HIV-coinfected individuals are more susceptible to progression to tuberculosis disease. We therefore hypothesized that HIV infection would potentially impair M. tuberculosis-specific protective immunity in individuals suffering from latent tuberculosis infection (LTBI) or active pulmonary tuberculosis (PTB). In this study, we demonstrated that M. tuberculosis/HIV-coinfected individuals have fewer circulating M. tuberculosis-specific CD4 T cells and that those that remained were functionally impaired in both LTBI and PTB settings. In addition, we showed that HIV infection significantly interferes with M. tuberculosis-induced systemic proinflammatory cytokine/chemokine responses. Taken together, these data suggest that HIV infection impairs functionally favorable M. tuberculosis-specific immunity.

## INTRODUCTION

Mycobacterium tuberculosis and type 1 human immunodeficiency virus (HIV-1) infections are coendemic in several regions of the world. In 2017, the World Health Organization (WHO) estimated that 1.7 billion individuals were infected with M. tuberculosis, among whom 9 to 11 million individuals suffered from tuberculosis disease (TB) (https://www.who.int/tb/publications/global_report/en/). Worldwide, about 1.3 million individuals were M. tuberculosis/HIV-1 coinfected (https://www.who.int/tb/publications/global_report/en/). The risk of developing TB increases throughout the course of HIV infection and with HIV/AIDS disease progression (1) and was estimated to be between 16- and 27-fold higher in HIV-infected individuals than in HIV-uninfected individuals (2–5).

These observations demonstrate that HIV-1 infection represents one of the major risk factors predisposing to TB development and suggest that protective M. tuberculosis-specific immunity is probably altered by HIV infection.

Although only partially defined (6), the protective components of M. tuberculosis-specific immunity include appropriate and efficient CD4 T-cell responses associated with type 1 cytokine secretion (gamma interferon [IFN-γ] and tumor necrosis factor alpha [TNF-α]), since IFN-γ receptor deficiency is associated with increased susceptibility to mycobacterial infections (7, 8) and anti-TNF-α therapy is associated with increased risk of TB reactivation (9).

HIV-1 infection triggers massive depletion of CD4 T cells (10), and it was first proposed that the increased risk to develop TB upon HIV infection might by associated with the severe reduction of CD4 T-cell numbers (11). In this context, several studies assessed the frequencies of M. tuberculosis-specific CD4 T cells in individuals suffering from latent tuberculosis infection (LTBI) or TB coinfected or not with HIV in the presence (12, 13) or absence (12–15) of conventional antiretroviral therapy (cART). The authors showed that HIV infection profoundly affects the frequencies and the differentiation profile of M. tuberculosis-specific CD4 T cells that cART initiation partially restored (12, 13), supporting the aforementioned hypothesis. However, long-term cART-treated and aviremic HIV-infected individuals remain at higher risk of developing TB than for HIV-uninfected individuals (16), suggesting that increased risk of developing TB in HIV-infected individuals might also be associated with M. tuberculosis-specific T-cell impairment and/or altered M. tuberculosis-specific innate immunity.

Antigen-specific T-cell impairment has been described in the context of chronic viral infections (17–19) or cancer (20) and is defined by a progressive loss of T-cell functions, including T-cell proliferation, cytokine production, and cytotoxic capacity (21). In this study, we hypothesized that progression from LTBI to PTB might be due not only to CD4 T-cell depletion but also to M. tuberculosis-specific CD4 T-cell functional impairment and/or altered M. tuberculosis-specific innate immunity. To test this hypothesis, M. tuberculosis-specific CD4 and CD8 T-cell frequencies and cytokine profiles were investigated in untreated Tanzanian individuals with LTBI or PTB and compared to those of untreated M. tuberculosis/HIV-coinfected individuals suffering from LTBI or PTB.

## RESULTS

The present study aimed to determine the influence of HIV infection on M. tuberculosis-specific T-cell responses in individuals with LTBI or PTB. Therefore, we analyzed the cytokine profile and cell lineage T-cell transcription factor expression together with the differentiation profile and the level of PD-1 expression of M. tuberculosis-specific CD4 and CD8 T cells in 112 individuals recruited from Tanzania (Table 1). The volunteers were screened for M. tuberculosis and HIV infection status and stratified into four groups: (i) HIV-uninfected individuals with LTBI (referred to as individuals with LTBI; *n* = 20), (ii) HIV-infected individuals with LTBI (referred to as individuals with HIV/LTBI; *n* = 15), (iii) HIV-uninfected individuals with PTB (referred to as individuals with PTB; *n* = 67), and (iv) HIV-infected individuals with PTB (referred to as individuals with HIV/PTB; *n* = 10).

**TABLE 1:**
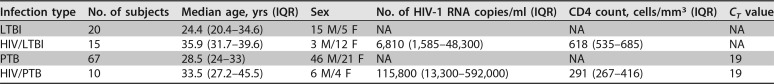
Demographic and clinical data^*a*^

a LTBI, latent tuberculosis infection; HIV, human immunodeficiency virus; PTB, pulmonary tuberculosis; M, male; F, female; NA, not applicable; IQR, interquartile range; *C_T_*, threshold cycle.

### HIV infection influences M. tuberculosis-specific CD4 T-cell frequencies and cytokine profiles.

**FIG 1:**
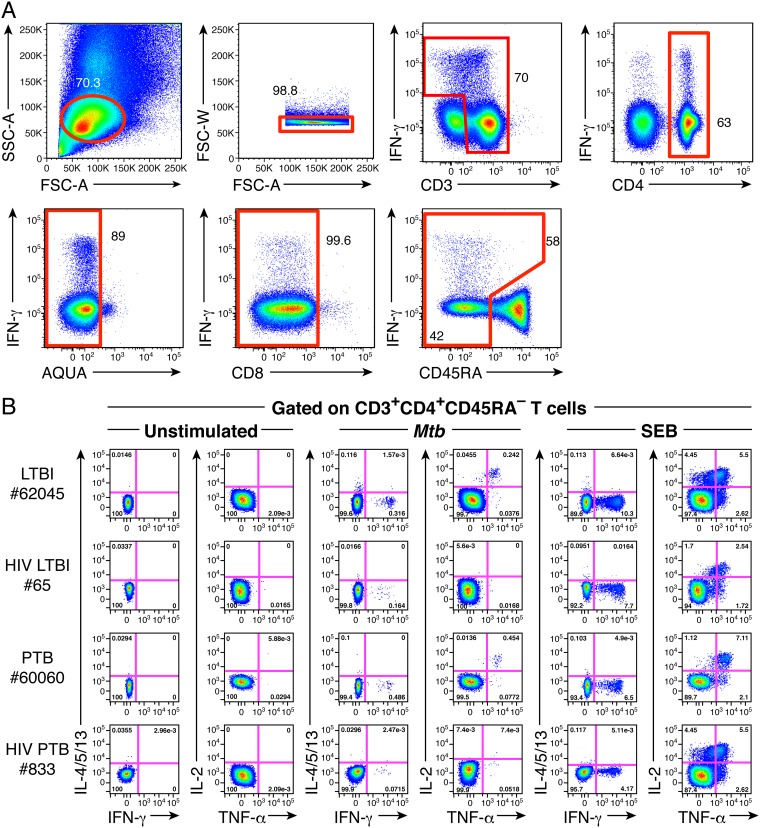
Assessment of M. tuberculosis-specific CD4 T-cell responses. (A) Gating strategy used to assess cytokine-producing CD4 T cells. (B) Representative flow cytometry profile of M. tuberculosis-specific CD4 T cells producing IFN-γ, IL-4/5/13, TNF-α, and/or IL-2 of individuals with LTBI (62045), HIV/LTBI (65), PTB (60060), or HIV/PTB (833). Cytokine profiles of CD4 T cells stimulated with SEB (positive control) or left unstimulated (negative control) are also shown.

The frequencies and functional profiles of M. tuberculosis-specific CD4 T-cell responses were assessed by intracellular cytokine staining (ICS) according to the gating strategy shown in Fig. 1A. In particular, the ability of antigen-specific CD4 T cells to produce IFN-γ, TNF-α, IL-2, IL-4, IL-5, and/or IL-13 in response to M. tuberculosis (ESAT-6 and CFP-10 peptide pools) or HAd5 (hexon-derived overlapping peptide pool) antigen-specific stimulation was assessed by multiparametric flow cytometry in 20 LTBI and 67 PTB individuals and compared to that in 15 HIV/LTBI- and 8 HIV/PTB-coinfected individuals. Of note, Th2 cytokines, i.e., IL-4, IL-5, and IL-13, were all assessed in the same flow cytometry fluorescence channel, which allowed the assessment of total Th2 cytokine production but prevented direct identification of individual IL-4, IL-5, or IL-13 antigen-specific CD4 T-cell responses.

**FIG 2:**
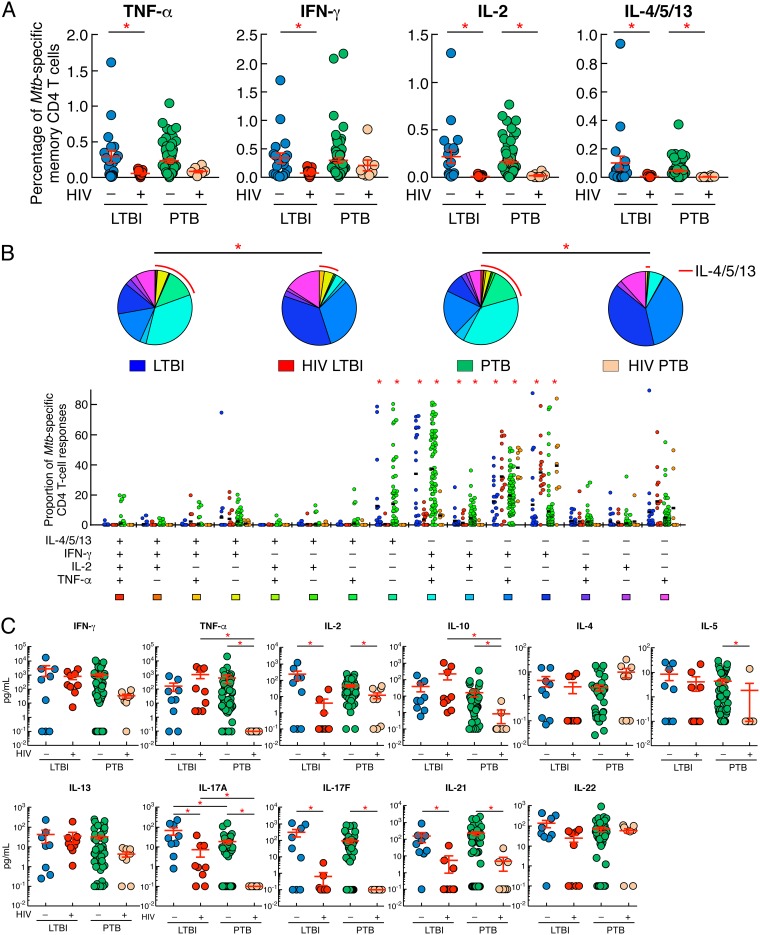
HIV infection influences M. tuberculosis-specific CD4 T-cell frequencies and cytokine profiles. (A) Percentage of M. tuberculosis-specific CD4 T cells producing TNF-α, IFN-γ, IL-2, or IL-4/5/13 of individuals with LTBI (*n* = 20), HIV/LTBI (*n* = 15), PTB (*n* = 67), or HIV/PTB (*n* = 8). (B) Proportion of M. tuberculosis-specific CD4 T-cell populations producing IFN-γ, IL-4/5/13, TNF-α, and/or IL-2 of individuals with LTBI (*n* = 20), HIV/LTBI (*n* = 15), PTB (*n* = 67), or HIV/PTB (*n* = 8). All the possible combinations of the responses are shown on the *x* axis, and the percentages of the functionally distinct cell populations within the M. tuberculosis-specific CD4 T-cell populations are shown on the *y* axis. Responses are grouped and color-coded on the basis of the number of functions. The pie chart summarizes the data, and each slice corresponds to the fraction of M. tuberculosis-specific CD4 T cell response with a given number of functions within the responding CD4 T-cell population. Bars correspond to the fractions of different functionally distinct CD4 T-cell populations within the total CD4 T cells. The red arc corresponds to IL-4/5/13-producing CD4 T-cell populations. (C) Levels of IFN-γ, TNF-α, IL-2, IL-4, IL-5, IL-13, IL-10, IL-17A, IL-17F, IL-21, and IL-22 produced in M. tuberculosis-stimulated culture supernatants of individuals with LTBI (*n* = 9), HIV/LTBI (*n* = 9), PTB (*n* = 50), or HIV/PTB (*n* = 8) assessed by multiplex bead array analyses (Luminex). Undetectable values were arbitrarily defined as 0.1 pg/ml. Individuals were color coded (A to C); Individuals with LTBI, blue; individuals with HIV/LTBI, red; individuals with PTB, green and individuals with HIV/PTB, orange. Red asterisks indicate statistical significance. Statistical significance (*P < *0.05) was calculated using one-way ANOVA (Kruskal-Wallis test) followed by a Mann-Whitney test (A and C). Statistical analyses of the global cytokine profiles (pie charts) (B) were performed by partial permutation tests using the SPICE software as described previously (54).

Cytokine profiles of M. tuberculosis-specific CD4 T cells from representative individuals with LTBI, HIV/LTBI, PTB, and HIV/PTB are shown in Fig. 1B. We first compared the frequencies of cytokine-producing M. tuberculosis-specific memory CD4 T cells from individuals with LTBI or PTB versus coinfected individuals, i.e., those with HIV/LTBI or HIV/PTB, respectively (Fig. 2A). The cumulative data showed that TNF-α-, IFN-γ-, IL-2-, or IL-4/5/13-producing M. tuberculosis-specific memory CD4 T-cell frequencies were significantly reduced in individuals with HIV/LTBI compared to individuals with LTBI (*P < *0.05) (Fig. 2A). In addition, IL-2- or IL-4/5/13-producing M. tuberculosis-specific memory CD4 T-cell frequencies were significantly reduced in individuals with HIV/PTB compared to individuals with PTB (*P < *0.05), while IFN-γ- or TNF-α-producing M. tuberculosis-specific memory CD4 T-cell frequencies were not significantly different between individuals with PTB coinfected or not with HIV (*P = *0.3744 and *P = *0.1096, respectively) (Fig. 2A). Interestingly, no significant differences were observed for TNF-α-, IFN-γ-, IL-2-, or IL-4/5/13-producing M. tuberculosis-specific memory CD4 T-cell frequencies between individuals with LTBI and PTB or between individuals with HIV/LTBI and HIV/PTB (*P > *0.05) (Fig. 2A).

We next analyzed the cytokine profile of M. tuberculosis-specific memory CD4 T cells of individuals with LTBI, LTBI/HIV, PTB, and HIV/PTB (Fig. 2B, pie charts). The cytokine profiles of M. tuberculosis-specific memory CD4 T cells of individuals with LTBI or PTB were significantly different from those of individuals with HIV/LTBI or HIV/PTB, respectively (*P < *0.05) (Fig. 2B, pie charts). Again, no significant differences were observed between individuals with LTBI and PTB or between individuals with HIV/LTBI and HIV/PTB (*P > *0.05) (Fig. 2B, pie charts).

In-depth analysis showed that M. tuberculosis-specific CD4 T-cell responses of individuals with LTBI or PTB were significantly enriched in polyfunctional IFN-γ^+^ IL-2^+^ TNF-α^+^ IL-4/5/13^−^ CD4 T cells (triple IFN-γ/IL-2/TNF-α^+^ M. tuberculosis-specific CD4 T cells), dual IFN-γ/IL-2^+^ CD4 T cells, and IFN-γ^−^ IL-2^−^ TNF-α^−^ IL-4/5/13^+^ CD4 T-cell populations (single IL-4/5/13^+^ M. tuberculosis-specific CD4 T cells) compared to those of individuals with HIV/LTBI or HIV/PTB (*P < *0.05) (Fig. 2B). In contrast, M. tuberculosis-specific CD4 T-cell responses of individuals with HIV/LTBI or HIV/PTB were significantly enriched in IFN-γ^+^ IL-2^−^ TNF-α^−^ IL-4/5/13^−^ CD4 T-cell populations (single IFN-γ^+^ M. tuberculosis-specific CD4 T cells) and IFN-γ^+^ IL-2^−^ TNF-α^+^ IL-4/5/13^−^ (dual IFN-γ/TNF-α^+^ M. tuberculosis-specific CD4 T cells) compared to those of individuals with LTBI or with PTB (*P < *0.05) (Fig. 2B). No significant differences were observed among cytokine-producing M. tuberculosis-specific memory CD4 T-cell frequencies between individuals with LTBI and PTB or between individuals with HIV/LTBI and HIV/PTB (*P > *0.05) (Fig. 2B).

To further characterize the cytokine profile of M. tuberculosis-specific T-cells, multiplex bead array analyses were performed on supernatants collected from ESAT-6/CFP-10 peptide pool (M. tuberculosis)-stimulated cell cultures. The cumulative data showed that M. tuberculosis-stimulated cell culture supernatants of individuals with HIV/LTBI or HIV/PTB secreted significantly lower levels of IL-2, IL-17A, IL-17F, and IL-21 (*P < *0.05) than M. tuberculosis-stimulated cell culture supernatants of individuals with LTBI or with PTB, respectively (Fig. 2C). In addition, M. tuberculosis-stimulated cell culture supernatants of individuals with HIV/PTB secreted significantly lower levels of TNF-α, IL-5, and IL-10 (*P < *0.05) and reduced levels of IL-13 (borderline significance; *P = *0.13) than M. tuberculosis-stimulated cell culture supernatants of individuals with PTB (Fig. 2C). Interestingly, M. tuberculosis-stimulated cell culture supernatants of individuals with PTB secreted significantly lower levels of IL-17A than cell culture supernatants individuals with LTBI (*P < *0.05) (Fig. 2C).

Taken together, our data indicate that HIV infection strongly influences M. tuberculosis-specific memory CD4 T-cell frequencies and cytokine profile, among which IL-2, IL-17A/F, and IL-21 production/secretion capacity appeared to be the most impacted, independently of M. tuberculosis disease status.

### HIV infection significantly influences Gata-3, T-bet, and RORγt expression.

Since HIV infection significantly influenced Th1, Th2, and Th17 cytokine production/secretion, we then determined whether HIV infection was associated with changes in the expression of Th1-, Th2-, and Th17-specific cell lineage transcription factors T-bet, Gata-3, and RORγt, respectively (22–24).

**FIG 3:**
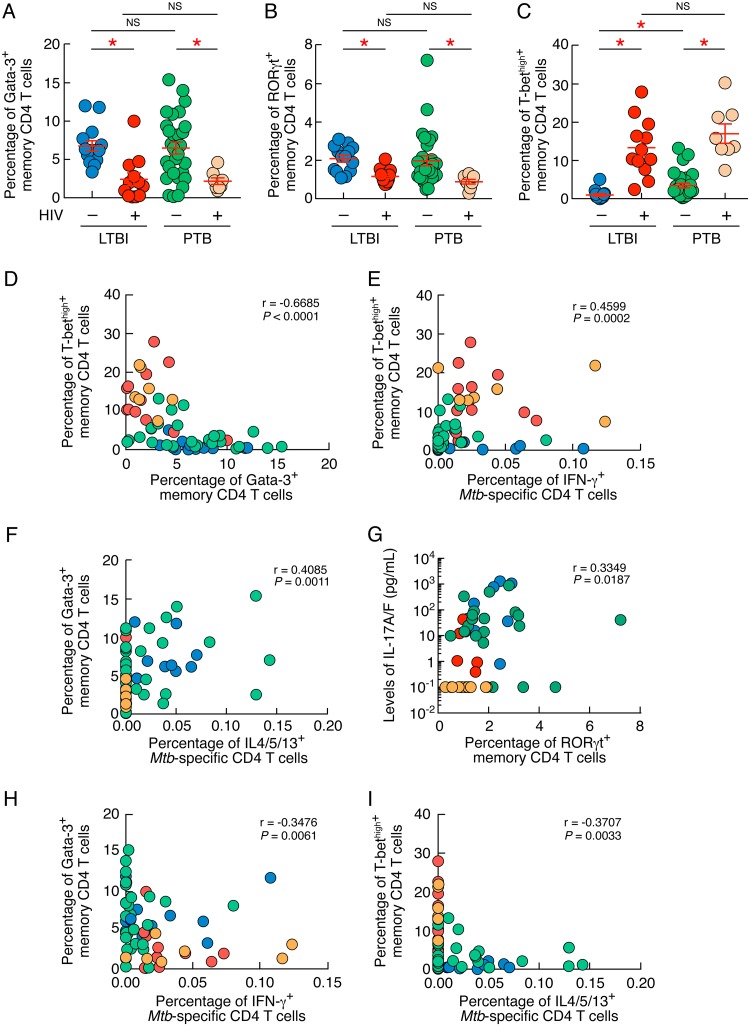
HIV infection significantly influences Gata-3, T-bet, and RORγt expression. (A to C) Percentages of memory (CD45RA^−^) CD4 T cells isolated from individuals with LTBI (*n* = 14), HIV/LTBI (*n* = 12), PTB (*n* = 29), or HIV/PTB (*n* = 8) expressing Gata-3 (A), RORγt (B), or T-bet^high^ (C). (D) Correlation between the percentage of memory CD4 T cells expressing T-bet^high^ and the percentage of memory CD4 T cells expressing Gata-3 in individuals with LTBI (*n* = 14), HIV/LTBI (*n* = 12), PTB (*n* = 26), or HIV/PTB (*n* = 8). (E) Correlation between the percentage of IFN-γ-producing M. tuberculosis-specific CD4 T cells and the percentage of memory CD4 T cells expressing T-bet^high^ of individuals with LTBI (*n* = 14), HIV/LTBI (*n* = 12), PTB (*n* = 29), or HIV/PTB (*n* = 8). (F) Correlation between the percentage of IL-4/5/13-producing M. tuberculosis-specific CD4 T cells and the percentage of memory CD4 T cells expressing Gata-3 of TB patients from individuals with LTBI (*n* = 14), HIV/LTBI (*n* = 12), PTB (*n* = 29), or HIV/PTB (*n* = 8). (G) Correlation between the levels of IL-17A/F detected in M. tuberculosis-stimulated culture supernatants and the percentage of memory CD4 T cells expressing RORγt individuals of individuals with LTBI (*n* = 9), HIV/LTBI (*n* = 6), PTB (*n* = 26), or HIV/PTB (*n* = 8). (H) Correlation between the percentage of memory CD4 T cells expressing Gata-3 and the percentage of M. tuberculosis-specific CD4 T cells producing IFN-γ in individuals with LTBI (*n* = 14), HIV/LTBI (*n* = 12), PTB (*n* = 26), or HIV/PTB (*n* = 8). (I) Correlation between the percentage of T-bet^high^ and the percentage of M. tuberculosis-specific CD4 T cells producing IL-4/5/13 in individuals with LTBI (*n* = 14), HIV/LTBI (*n* = 12), PTB (*n* = 26), or HIV/PTB (*n* = 8). Statistical significance (*; *P < *0.05) was calculated using one way Anova (Kruskal-Wallis test) followed by a Mann-Whitney test (A to C) or Spearman rank test for correlations (D to I). NS, not significant.

The combined data showed that the percentages of memory CD4 T cells expressing Gata-3 or RORγt were significantly reduced in individuals with HIV/LTBI or HIV/PTB compared to those in individuals with LTBI or PTB (Gata-3, 2.4% and 2% versus 6.7% and 6.4%, respectively [*P < *0.05]; RORγt, 1.1% and 0.8% versus 2% and 1.9%, respectively [*P < *0.05]) (Fig. 3A and B). In contrast, the percentage of memory CD4 T cells expressing high levels of T-bet (T-bet^high^) was significantly increased in individuals with HIV/LTBI or HIV/PTB compared to that in individuals with LTBI or PTB (13% and 17% versus 0.9% and 3.4%, respectively [*P < *0.05]) (Fig. 3C). Notably, the frequencies of memory CD4 T cells expressing T-bet^high^ were significantly higher in individuals with PTB than in individuals with LTBI (3.4% versus 0.9% [*P < *0.05]) (Fig. 3C). Interestingly, the percentage of T-bet^high^ memory CD4 T cells was inversely correlated with the percentage of memory CD4 T cells expressing Gata-3 (*r* = −0.6685; *P < *0.0001) (Fig. 3D), supporting previous observations (25).

We then determined whether the M. tuberculosis-specific CD4 T-cell cytokine profiles observed in individuals with LTBI or PTB coinfected or not with HIV were associated with T-bet, Gata-3, or RORγt expression profiles. To address this issue, we plotted the percentage of M. tuberculosis-specific CD4 T cells producing IFN-γ or IL-4/5/13 against the percentage of memory CD4 T cells expressing T-bet^high^ or Gata-3 from the same patients (Fig. 3E and F) or the levels of IL-17A/F detected in M. tuberculosis-stimulated cell culture supernatants against the percentage of memory CD4 T cells expressing RORγt from the same patients (Fig. 3G). The cumulative data demonstrate that the percentage of IFN-γ-producing M. tuberculosis-specific CD4 T cells directly correlated with the percentage of T-bet^high^ memory CD4 T cells (*r* = 0.4599; *P = *0.0002) (Fig. 3E) and the percentage of IL-4/5/13-producing M. tuberculosis-specific CD4 T cells directly correlated with the percentage of memory CD4 T cells expressing Gata-3 (*r* = 0.4085; *P = *0.0011) (Fig. 3F). In addition, the levels of IL-17A/F detected in M. tuberculosis-stimulated cell culture supernatant directly correlated with the percentage of memory CD4 T cells expressing RORγt from the same patients (*r* = 0.3349; *P = *0.0187) (Fig. 3G). Notably, the percentages of Gata-3 and T-bet^high^ memory CD4 T cells negatively correlated with the percentage of M. tuberculosis-specific CD4 T cells producing IFN-γ and IL-4/5/13, respectively (Gata-3 versus IFN-γ, *r* = −0.3707 and *P < *0.05; T-bet^high^ versus IL-4/5/13, *r* = −0.3476 and *P < *0.05) (Fig. 3H and I).

Taken together, these data indicate that HIV infection strongly influenced Gata-3, RORγt, and T-bet^high^ T-cell lineage transcription factor expression profiles. In particular, HIV coinfection resulted in a significant shift of the M. tuberculosis-specific cytokine profile from a mixed Th1/Th2/Th17 cytokine profile associated with increased Gata-3 and RORγt and reduced T-bet^high^ expression observed in individuals with LTBI or PTB to a Th1-restricted cytokine profile associated with increased T-bet^high^ and reduced Gata-3 or RORγt expression observed in individuals with HIV/LTBI or HIV/PTB.

### HIV infection influences PD-1 expression on M. tuberculosis-specific CD4 T cells.

Our data showed that HIV infection significantly reduced IL-2 production/secretion from M. tuberculosis-specific CD4 T cells. Since IL-2 production/secretion capacity might be reduced in effector memory cells (EM; CD45RA^−^ CCR7^−^) and/or by coinhibitory molecule expression (26, 27), we therefore assessed whether HIV infection might have influenced CCR7 and/or PD-1 surface expression on M. tuberculosis-specific CD4 T cells.

**FIG 4:**
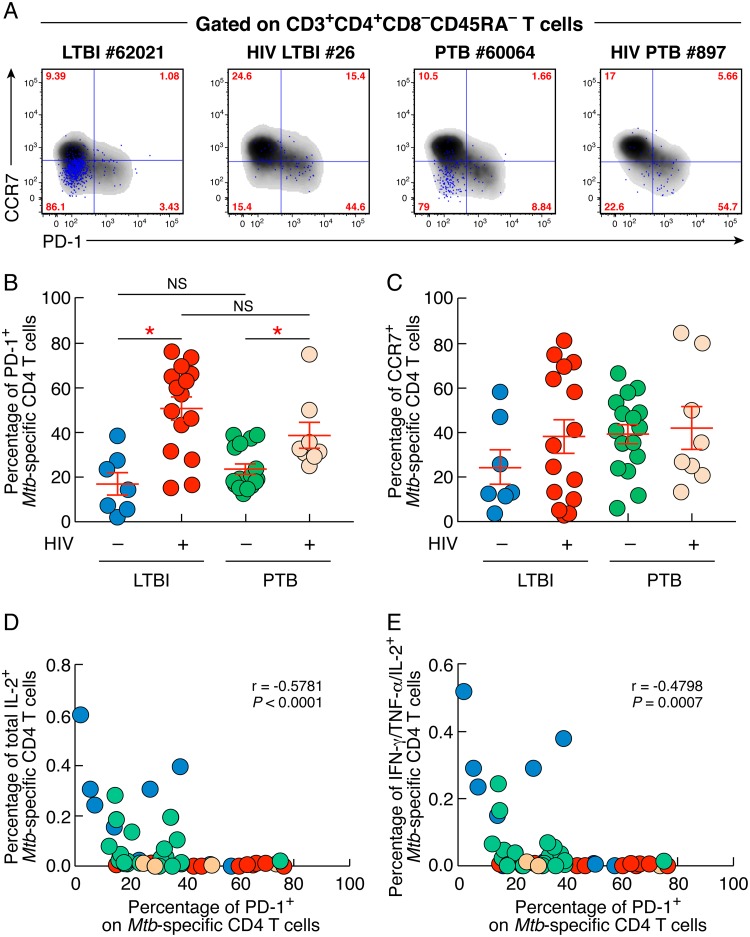
HIV infection influences PD-1 expression on M. tuberculosis-specific CD4 T cells. (A) Representative flow cytometry profile of M. tuberculosis-specific memory (CD45RA^−^) CD4 T cells (blue dots) isolated from one individual with LTBI (62021), HIV/LTBI (26), PTB (60064), or HIV/PTB (897) expressing PD-1 and/or CCR7. (B and C) Percentages of M. tuberculosis-specific CD4 T cells isolated from individuals with LTBI (*n* = 7), HIV/LTBI (*n* = 15), PTB (*n* = 16), or HIV/PTB (*n* = 8) expressing PD-1 (B) and/or CCR7 (C). (D and E) Correlation between the percentage of M. tuberculosis-specific CD4 T cells expressing PD-1 and the percentage of total IL-2-producing M. tuberculosis-specific memory CD4 T cells (D) or the percentage of IFN-γ/IL-2/TNF-α-producing M. tuberculosis-specific memory CD4 T cells of individuals with LTBI (*n* = 7), HIV/LTBI (*n* = 15), PTB (*n* = 16) or HIV/PTB (*n* = 8) (E). Statistical significance (*; *P < *0.05) was calculated using one-way ANOVA (Kruskal-Wallis test) followed by the Mann-Whitney test (B and C) or Spearman rank test for correlation (D and E).

The percentage of M. tuberculosis-specific memory CD4 T cells expressing PD-1 was significantly increased in individuals with HIV/LTBI and HIV/PTB compared to individuals with LTBI and PTB, respectively (51% and 38% versus 17% and 24%; *P < *0.05) (Fig. 4A and B). In contrast, the percentages of M. tuberculosis-specific memory CD4 T cells expressing CCR7 did not differ between individuals with HIV/LTBI or HIV/PTB and individuals with LTBI or PTB (*P < *0.05) (Fig. 4A and C).

We then investigated whether the M. tuberculosis-specific CD4 T-cell cytokine profiles observed in individuals with LTBI or PTB coinfected or not with HIV were associated with PD-1 expression. To address this issue, we plotted the proportion of M. tuberculosis-specific CD4 T cells producing IL-2 (either total IL-2-producing M. tuberculosis-specific memory CD4 T cells or IFN-γ/IL-2/TNF-α-producing M. tuberculosis-specific memory CD4 T cells) against the percentage of M. tuberculosis-specific memory CD4 T cells expressing PD-1 from the same patients (Fig. 4D and E). The cumulative data showed that the percentage of M. tuberculosis-specific CD4 T cells expressing PD-1 inversely correlated with the proportion of IL-2-producing M. tuberculosis-specific memory CD4 T cells (*r* = −0.5781; *P < *0.0001) (Fig. 4D) or the proportion of IFN-γ/IL-2/TNF-α-producing M. tuberculosis-specific memory CD4 T cells (*r* = −0.4798; *P = *0.0007) (Fig. 4E).

These data indicate that PD-1 expression on M. tuberculosis-specific CD4 T cells is induced by HIV infection and associated with reduced IL-2 production/secretion by M. tuberculosis-specific CD4 T cells.

### HIV infection did not influence HAd5-specific CD4 T-cell frequencies, cytokine profiles, or PD-1 expression.

**FIG 5:**
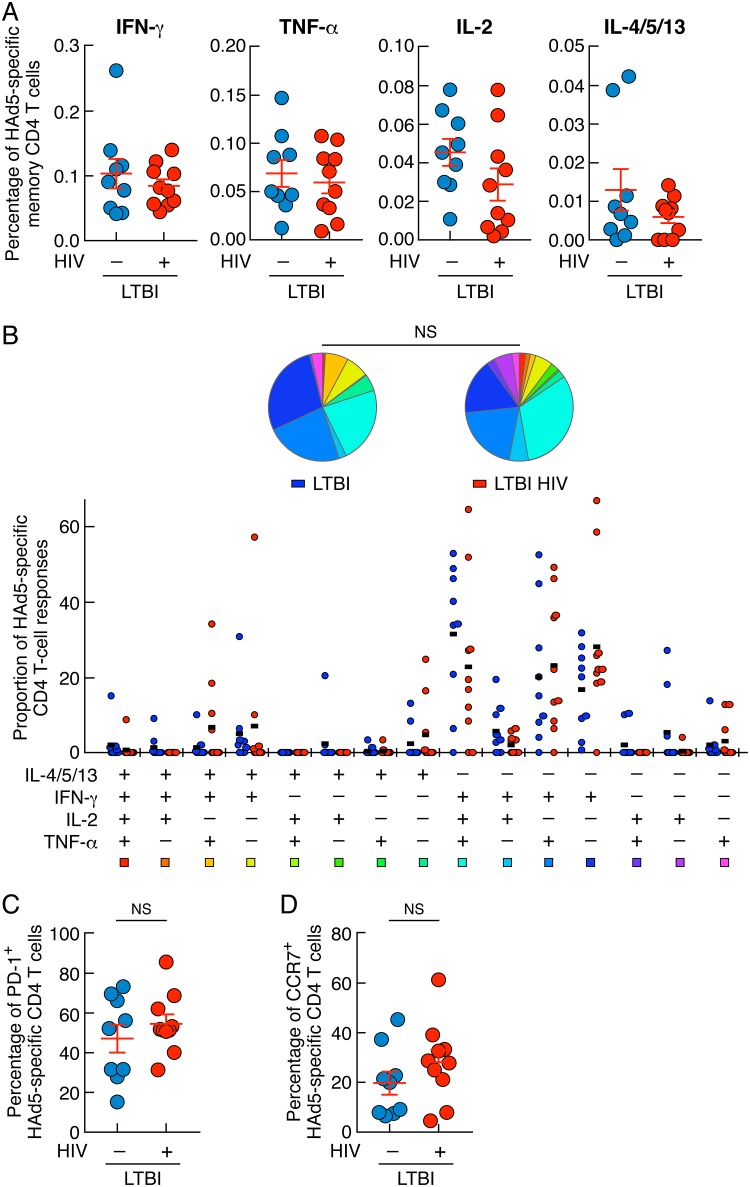
HIV infection did not influence HAd5-specific CD4 T-cell frequencies, cytokine profiles, or PD-1 expression. (A) Percentages of HAd5-specific CD4 T cells producing IFN-γ, TNF-α, IL-2, or IL-4/5/13 of individuals with LTBI (*n* = 9) or HIV/LTBI (*n* = 10). (B) Proportion of HAd5-specific CD4 T-cell populations producing IFN-γ, IL-4/5/13, TNF-α, and/or IL-2 of individuals with LTBI (*n* = 9) or HIV/LTBI (*n* = 10). All the possible combinations of the responses are shown on the *x* axis, and the percentages of the functionally distinct cell populations within the HAd5-specific CD4 T-cell populations are shown on the *y* axis. Responses are grouped and color-coded on the basis of the number of functions. The pie chart summarizes the data, and each slice corresponds to the fraction of HAd5-specific CD4 T cell response with a given number of functions within the responding CD4 T-cell population. Bars correspond to the fractions of different functionally distinct CD4 T-cell populations within the total CD4 T cells. (C and D) Percentages of HAd5-specific CD4 T cells isolated from individuals with LTBI (*n* = 9) or HIV/LTBI (*n* = 10) expressing PD-1 (C) and/or CCR7 (D). Statistical significance (*; *P < *0.05) was calculated using the Mann-Whitney test (A, C, and D). Statistical analyses of the global cytokine profiles (pie charts [B]) were performed by partial permutation tests using the SPICE software as described previously (54).

In order to determine whether HIV infection specifically influenced M. tuberculosis-specific CD4 T-cell responses in the present study, HAd5-specific CD4 T-cell responses were also assessed in LTBI with or without HIV coinfection. The cumulative data indicated that the frequencies and cytokine profiles of HAd5-specific CD4 T cells were not significantly influenced by HIV infection (*P > *0.05) (Fig. 5A and B). In addition, the percentages of HAd5-specific CD4 T cells expressing PD-1 and/or CCR7 did not significantly differ between individuals with LTBI and individuals with HIV/LTBI (*P > *0.05) (Fig. 5C and D). Taken together, these data indicated that in contrast to the case with M. tuberculosis-specific CD4 T cells, HIV infection influenced neither HAd5-specific CD4 T-cell frequencies nor their cytokine profiles or PD-1 expression.

### HIV infection influences IL-2 production from M. tuberculosis-specific CD8 T-cell responses.

**FIG 6:**
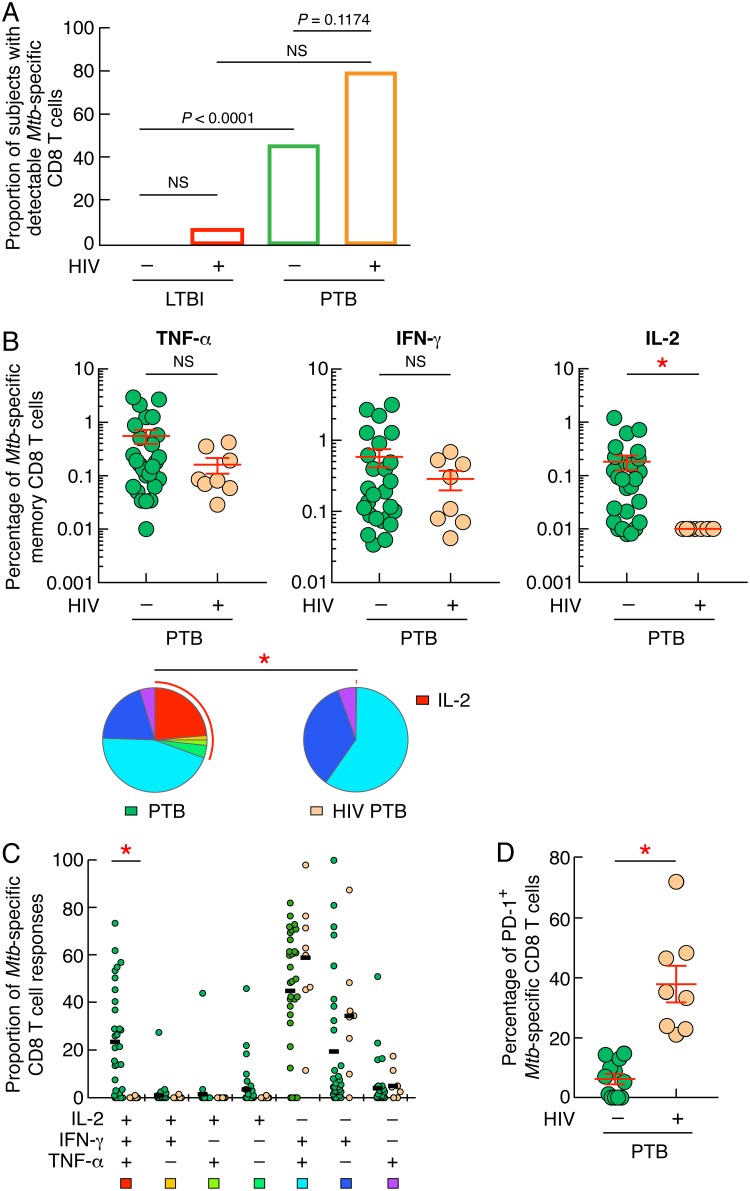
HIV infection influences IL-2 production from M. tuberculosis-specific CD8 T-cell responses. (A) Proportions of subjects with detectable M. tuberculosis-specific CD8 T cells. (B) Percentages of M. tuberculosis-specific CD8 T cells producing TNF-α, IFN-γ, and/or IL-2 of individuals with PTB (*n* = 31) or HIV/PTB (*n* = 8). Undetectable values were arbitrarily defined as 0.01%. (C) Cytokine profile of M. tuberculosis-specific CD8 T cells of individuals with PTB (*n* = 31) or HIV/PTB (*n* = 8). The red arc identifies IL-2-producing cell populations (C). (D) Percentage of M. tuberculosis-specific CD8 T cells expressing PD-1 of individuals with PTB (*n* = 12) or HIV/PTB (*n* = 8). Statistical significance (*; *P < *0.05) was calculated using chi-square test (A) and Mann-Whitney test (B and D). Statistical analyses of the global cytokine profiles (pie charts [B] and panel C) were performed by partial permutation tests using the SPICE software as described previously (54).

M. tuberculosis-specific CD8 T cells are more frequently detected in PTB than in LTBI (25, 28, 29). However, the impact of HIV infection on M. tuberculosis-specific CD8 T-cell responses remained to be addressed. We therefore investigated whether HIV infection might influence the proportion of individuals with detectable M. tuberculosis-specific CD8 T cells and the cytokine profile of M. tuberculosis-specific CD8 T cells of individuals with LTBI or PTB coinfected or not with HIV. The ability of M. tuberculosis-specific CD8 T cells to produce IFN-γ, TNF-α, and/or IL-2 was assessed in 20 LTBI and 67 PTB individuals and compared to that of CD8 T cells from 15 HIV/LTBI- and 10 HIV/PTB-coinfected individuals by flow cytometry. As shown in Fig. 6A, the proportions of subjects with detectable M. tuberculosis-specific CD8 T cells did not significantly differ between individuals with HIV/LTBI or HIV/PTB and individuals with LTBI or PTB, respectively (7% versus 0% and 80% versus 46%; *P > *0.05) (Fig. 6A). Due to the limited number of LTBI and HIV/LTBI subjects with detectable M. tuberculosis-specific CD8 T cells (Fig. 6A), M. tuberculosis-specific CD8 T-cell frequencies, cytokine profile and phenotype were restricted to individuals with PTB (Fig. 6B). The cumulative data indicated that the frequencies of IL-2-producing M. tuberculosis-specific CD8 T cells were significantly reduced in individuals with HIV/PTB compared to individuals with PTB (*P < *0.05), while frequencies of IFN-γ- or TNF-α-producing M. tuberculosis-specific CD8 T cells did not significantly differ between individuals with PTB and individuals with HIV/PTB (*P = *0.7202 and *P = *0.4585, respectively) (Fig. 6B). The cytokine profiles of M. tuberculosis-specific CD8 T-cell responses in PTB versus HIV/PTB differed significantly (*P < *0.05) (Fig. 6C, pie charts). A significant loss of polyfunctional IFN-γ^+^ IL-2^+^ TNF-α^+^-producing M. tuberculosis-specific CD8 T cells in individuals with HIV/PTB compared to individuals with PTB (0% versus 23%; *P < *0.05) (Fig. 6C) was observed.

Next we measured the PD-1 expression level of M. tuberculosis-specific CD8 T cells. The cumulative data indicate that PD-1 expression was significantly increased on M. tuberculosis-specific memory CD8 T cells in HIV/PTB compared to PTB (*P < *0.05) (Fig. 6D), indicating that HIV infection strongly influenced PD-1 expression on M. tuberculosis-specific CD8 T cells and was associated with reduced IL-2 production capacity.

### Influence of HIV replication on M. tuberculosis-specific CD4 T-cell cytokine profile and phenotype.

**FIG 7:**
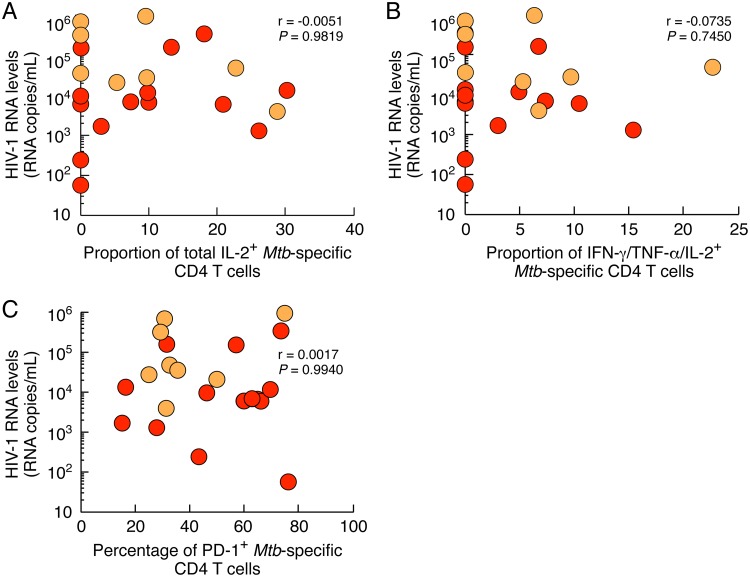
Influence of HIV replication on M. tuberculosis-specific CD4 T-cell cytokine profile and phenotype. Correlation between the levels HIV-1 RNA and the proportion of total IL-2-producing M. tuberculosis-specific memory CD4 T cells (A) or the proportion of IFN-γ/IL-2/TNF-α-producing M. tuberculosis-specific memory CD4 T cells (B) or the percentage of M. tuberculosis-specific CD4 T cells expressing PD-1 (C) of individuals HIV/LTBI (*n* = 15) or HIV/PTB (*n* = 8). Statistical significance (*; *P < *0.05) was calculated using the Spearman rank test for correlation.

Previous studies indicated that in HIV-infected individuals, the level of PD-1 expression was increased on HIV-specific T cells but not on cytomegalovirus (CMV)- or Epstein-Barr virus (EBV)-specific T cells (18, 19). In addition, PD-1 expression level directly correlated with HIV viral load, suggesting that excessive and continuous antigen stimulation functionally impaired HIV-specific T cells (17, 30). However, the impact of ongoing HIV replication on M. tuberculosis-specific CD4 T cells remains unclear. We then determined whether M. tuberculosis-specific CD4 T-cell cytokine profiles observed in M. tuberculosis/HIV-coinfected individuals were associated with HIV viral load. To address this issue, we plotted the proportion of M. tuberculosis-specific CD4 T cells producing IL-2 (either total IL-2-producing M. tuberculosis-specific memory CD4 T cells or IFN-γ/IL-2/TNF-α-producing M. tuberculosis-specific memory CD4 T cells) or expressing PD-1 against HIV viral load (Fig. 7). Our results show that the proportion of M. tuberculosis-specific CD4 T cells producing IL-2 or the percentage of M. tuberculosis-specific CD4 T cells expressing PD-1 did not correlate with HIV viral load (*r* = −0.0051, *r* = −0.0735, and *r* = −0.0017; *P > *0.05; respectively) (Fig. 7), suggesting that the degree of M. tuberculosis-specific CD4 T-cell impairment was independent of ongoing HIV replication.

### HIV infection dampens the level of systemic inflammation markers in PTB/HIV-coinfected individuals.

**FIG 8:**
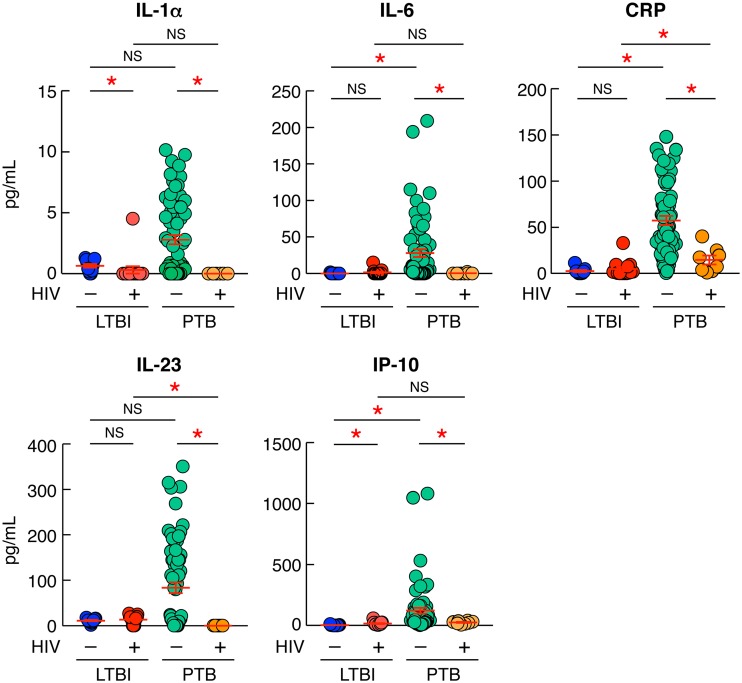
HIV infection dampens the level of systemic inflammation markers in PTB/HIV-coinfected individuals. Shown are serum levels of IL-1α, IL-6, CRP, IL-23, and IP-10 of individuals with LTBI (*n* = 11), HIV/LTBI (*n* = 15), PTB (*n* = 67), or HIV/PTB (*n* = 8). Statistical significance (*; *P < *0.05) was calculated using the Mann-Whitney test.

One objective of the present study was to determine whether HIV infection may influence systemic inflammation markers in the Tanzanian cohort studied. Hence, the serum levels of IL-1α, IL-6, C-reactive protein (CRP), IL-23, and IP-10 were assessed in individuals with LTBI or PTB coinfected or not with HIV using a multiplex bead assay (Fig. 8). Notably, the serum levels of IL-6, CRP, and IP-10 were significantly increased in individuals with PTB compared to individuals with LTBI or HIV/LTBI (*P < *0.05), supporting previous observations (31) (Fig. 8). Interestingly, the serum levels of IL-1α, IL-6, CRP, IL-23, and IP-10 were significantly reduced in individuals with HIV/PTB compared to individuals with PTB (*P < *0.05) (Fig. 8).

Taken together, these data suggest that HIV infection significantly interferes with M. tuberculosis-induced systemic proinflammatory cytokine/chemokine responses.

## DISCUSSION

Previous studies indicate that the cytokine profile and the frequencies of M. tuberculosis-specific CD4 T cells could be impacted by HIV coinfection (14, 15, 32) and that these responses were not fully restored under cART (12, 13). In the present study, we hypothesized that progression from LTBI to PTB might not be due to CD4 T-cell depletion only but might also be driven by M. tuberculosis-specific CD4 and CD8 T-cell functional impairment. To test this hypothesis, we assessed the cytokine profile, the transcription factor expression profile, and the phenotype of M. tuberculosis-specific T cells of untreated Tanzanian individuals suffering from LTBI or PTB and compared them to those of untreated M. tuberculosis/HIV-coinfected individuals suffering from LTBI or PTB.

We showed that HIV-infected individuals harbored reduced M. tuberculosis-specific CD4 T-cell frequencies associated with significant changes in M. tuberculosis-specific CD4 T-cell cytokine profiles. Interestingly, HIV infection did not influence all cytokines to the same extent. In particular, we showed that HIV infection significantly reduced the proportion of IL-2- or Th2 (IL-4/IL-5/IL-13)-producing M. tuberculosis-specific CD4 T cells in LTBI or PTB. These data, however, contrast with a recent study that did not report significant changes in IL-2 production although changes in M. tuberculosis-specific CD4 T-cell cytokine profiles were observed (15). These differences might be attributed at least in part to the cohort studied, i.e., South African versus Tanzanian individuals. Indeed, we recently observed that M. tuberculosis-specific CD4 T-cell cytokine profile of PTB patients from Tanzania were primarily composed of polyfunctional Th1 and Th2 cells, while M. tuberculosis-specific CD4 T-cell cytokine profiles of TB patients from South Africa were dominated by single IFN-γ and dual IFN-γ/TNF-α (25).

In addition, we showed that M. tuberculosis/HIV-coinfected individuals harbored reduced capacity to secrete IL-17A, IL-17F, and IL-21 cytokines following *in vitro* M. tuberculosis antigen stimulations, supporting previous observations for LTBI subjects (33) and suggesting that Th17 cells may contribute to control of M. tuberculosis replication.

Interpretation of cytokine production from stimulated cells remains challenging, especially within individuals with disease. Notably, the reduced cytokine levels observed in some individuals might be associated with reduced M. tuberculosis-specific T-cell frequencies and/or functional impairment. In addition, various parameters such as transcription factor expression (22, 23), memory subsets (26), and/or the expression of coinhibitory molecules (17–19, 27) could influence the cytokine profile of antigen-specific CD4 T cells. Here we demonstrate that changes of M. tuberculosis-specific CD4 T-cell cytokine profiles in M. tuberculosis/HIV-coinfected individuals was associated with an increase of T-bet and PD-1 expression accompanied by a reduction of Gata-3 and RORγt expression in the memory CD4 T cells. The proportion of IL-4/5/13-producing M. tuberculosis-specific CD4 T cells was inversely correlated with the percentage of memory CD4 T cells expressing T-bet^high^, and the proportion of IL-2-producing M. tuberculosis-specific CD4 T cells was inversely correlated with the percentage of PD-1-expressing M. tuberculosis-specific CD4 T cells. Consistent with previous studies, we did not observe any influence of HIV infection on the differentiation profile of M. tuberculosis-specific CD4 T cells (15, 34), indicating that HIV infection might influence the M. tuberculosis-specific CD4 T-cell cytokine profile by influencing the transcription factor profile and PD-1 expression. Several clinical studies performed in multidrug-resistant TB cases showed an increased rate of *Mycobacterium* clearance associated with radiological lung improvement when recombinant IL-2 was applied (35).

To determine whether HIV infection might specifically impact M. tuberculosis-specific CD4 T-cell responses, we also assessed HAd5-specific T-cell responses in LTBI with or without HIV coinfection in the same volunteers. HAd5 is an intracellular pathogen that usually causes upper and lower respiratory tract infections (36), controlled by efficient polyfunctional CD4 and CD8 T-cell responses (37–39), leading to persistent sub-clinical infections in most of immunocompetent individuals (40, 41). In contrast, in immunocompromised individuals, reactivation of HAd5 replication can lead to fatal disease progression (40). Interestingly, HAd5-specific CD4 T-cell frequencies, cytokine profiles, and phenotypes did not differ between HIV-uninfected and HIV-infected individuals, suggesting that HIV infection might specifically influence M. tuberculosis-specific CD4 T-cell responses. Notably, we cannot exclude that distinct scenario or set of circumstances were associated with these observations. However, neither the proportion of IL-2-producing M. tuberculosis-specific CD4 T cells nor the level of PD-1 on M. tuberculosis-specific CD4 T cells of M. tuberculosis/HIV-coinfected individuals correlated with HIV viral load, suggesting that the influence of HIV infection on M. tuberculosis-specific CD4 T-cell cytokine profile and phenotype might be indirect. The fact that the cytokine profiles of M. tuberculosis-specific CD8 T-cell responses in PTB versus HIV/PTB differed significantly provides additional evidence that HIV infection may substantially influence M. tuberculosis-specific T-cell responses during active TB disease and echoes the recent findings from Chiacchio et al. that showed that M. tuberculosis-specific CD8 T cells from M. tuberculosis/HIV-coinfected individuals are more monofunctional (42).

One potential mechanism by which HIV infection may influence TB disease progression and therefore M. tuberculosis-specific T-cell cytokine profile and phenotype might be through granuloma disorganization. Indeed, M. tuberculosis replication is partially contained by organized granulomas formed by macrophages and CD4 T cells in the lung tissues (1), which HIV infection may disrupt through CD4 T-cell depletion, favoring M. tuberculosis dissemination and extrapulmonary disease (43, 44).

Finally, during PTB, M. tuberculosis-exposed/infected lung macrophages produce large quantities of proinflammatory cytokines and chemokines involved in the chemoattraction of monocytes and T cells to the sites of infection (45, 46). These cytokines/chemokines are detectable in the sera of individuals with PTB, and the serum cytokine profile may be indicative of the level of systemic inflammation, antigen load, TB disease severity, and hospitalization duration (47). Therefore, in the present study, we were interested in how and if HIV infection may interfere with M. tuberculosis-induced cytokine/chemokine production. We compared the serum levels of proinflammatory cytokines and chemokines of individuals with LTBI and PTB coinfected or not with HIV. We found that the serum levels of IL-1α, IL-6, CRP, IL-23, and IP-10 were significantly reduced in HIV/PTB-coinfected individuals compared to the levels in individuals with PTB only, suggesting that HIV infection significantly dampens M. tuberculosis-induced systemic proinflammatory cytokine/chemokine response.

The mechanism by which HIV suppresses the M. tuberculosis-induced systemic proinflammatory cytokine response in PTB requires further investigation. However, one potential mechanism might be through M. tuberculosis-specific CD4 T-cell depletion. Indeed, M. tuberculosis-induced systemic proinflammatory cytokine response in PTB is mainly mediated by macrophages, epithelial cells, and fibroblasts in response to proinflammatory cytokines released by M. tuberculosis-specific CD4 T cells (48–50), which might be reduced in functionally impaired M. tuberculosis-specific CD4 T cells.

In conclusion, the present study provides evidence that M. tuberculosis-specific CD4 and CD8 T-cell responses are impacted by HIV coinfection, resulting in pronounced variations in the qualitative and quantitative profile of M. tuberculosis-specific T cells in human populations. The precise mechanism by which HIV infection interferes with M. tuberculosis-specific protective immunity still remains to be determined and probably involves both the depletion and the functional impairment of M. tuberculosis-specific T cells.

## MATERIALS AND METHODS

### Study group and cell isolation.

In total, 112 subjects participated to this study and were recruited at the Mwananyamala hospital, Dar es Salaam, and at the TB clinic of Bagamoyo (TZ) (Table 1). Pulmonary TB patients were selected based on sputum smear microscopy confirmed by GeneXpert assay, and HIV infection was defined based on a rapid serological test (Alere Determine HIV-1/2 test). Individuals with no sign of active TB infection but harboring CFP-10/ESAT-6 peptide pool-specific T-cell responses assessed by intracellular cytokine staining were classified as having LTBI. No statistical method was used to predetermine sample size. Plasma viral load (HIV-1 RNA) was assessed by COBAS AmpliPrep/TaqMan HIV-1 test (Roche, Switzerland) as previously described (51). Blood mononuclear cells were isolated as previously described (52).

### Ethics statement.

All participants were adults and provided written informed consent, and the study protocol was approved for TZ by the Ethikkomission beider Basel (EKBB; Basel, Switzerland; reference number 257/08), the Ifakara Health Institute Institutional Review Board, and the National Institute for Medical Research (NIMR; Dar es Salaam, United Republic of Tanzania; reference number NIMR/HQ/R.8a/Vol.IX/1098).

### Antibodies.

The following monoclonal antibodies (MAbs) were used in different combinations: CD3-allophycocyanin (APC)-H7 (clone SK7), CD4-PECF594 or CD4-APC (clone RPA-T4), CD8-PB (clone RPA-T8), IFN-γ–AF700 (clone B27), TNF-α–PeCy-7 (clone MAb11), IL-4–phycoerythrin (PE) (clone 3010.211), Gata-3–PeCy–7 (clone L50-823), CCR7-fluorescein isothiocyanate (FITC) (clone 3D12), and CD8-BV605 (clone SK1), all from Becton, Dickinson (BD); CD45RA-BV711 (clone HI100), IL-2–peridinin chlorophyll protein (PerCp)–Cy5.5 (clone MQ1-17H12), IL-5–PE (clone TRFK5), IL-13–PE (clone JES10-5A2), T-bet–PerCp–Cy5.5 (clone 4B10), and PD-1–BV421 (clone EH12.2H7), purchased from BioLegend; and RORγt-PE (clone AFKJS-9) from eBioscience.

### Antigens.

M. tuberculosis-derived CFP-10 and ESAT-6 peptide pools were composed of 15-mers overlapping by 11 amino acids encompassing the entire sequences of the proteins, and all peptides were purified by high-performance liquid chromatography (HPLC) (>90% purity). In some experiments, cells were also stimulated with 1 μg of HAd5 hexon-derived overlapping peptide pool (Miltenyi).

### *Ex vivo* assessment of CD4 and CD8 T-cell cytokine profile by intracellular cytokine staining.

Peripheral blood mononuclear cells (PBMCs) were stimulated overnight in complete medium (RPMI [Invitrogen], 10% fetal calf serum [FCS; Invitrogen], 100 μg/ml of penicillin, 100 U/ml of streptomycin [BioConcept]) with ESAT-6 and CFP-10 peptide pools (1 μg/ml) or with *Staphylococcus* enterotoxin B (SEB; 250 ng/ml) or with HAd5 hexon-derived overlapping peptide pool, or they were left unstimulated in the presence of Golgiplug (1 μl/ml; BD) as previously described (53). At the end of the stimulation period, cells were washed and stained (20 min at 4°C) for dead cells using the aqua LIVE/DEAD stain kit (Invitrogen), washed, and stained (20 min at 4°C) with MAbs to CD3, CD4, CD8, PD-1, CCR7, and CD45RA. Cells were then permeabilized (30 min at 20°C) (Cytofix/Cytoperm; BD) and stained (20 min at 20°C) with MAbs to TNF-α, IFN-γ, IL-2, IL-4, IL-5, and IL-13. The criterion for scoring antigen-specific CD4 or CD8 T-cell responses as positive was as follows: the cytokine (IFN-γ, TNF-α, or IL-2) frequency obtained in the sample had to exceed 0.03% after background subtraction.

### Assessment of T-bet, Gata-3, and RORγt expression.

PBMCs were washed, stained (20 min at 4°C) for dead cells using the aqua LIVE/DEAD stain kit, and then washed and stained (20 min at 4°C) for CD3, CD4, CD8, and CD45RA. Cells were then washed, permeabilized (45 min at 4°C) (Foxp3 fixation/permeabilization kit; eBioscience), and stained (20 min at 4°C) with MAbs to T-bet, RORγt, and Gata-3.

### Assessment of the cytokine profile of M. tuberculosis-stimulated culture supernatants by multiplex bead assay.

PBMCs (2 × 10^5^ cells) were stimulated for 24 h in complete medium with ESAT-6 and CFP-10 peptide pools (1 μg/ml) or with SEB (250 ng/ml) or were left unstimulated (negative control). At the end of the stimulation period, culture supernatants were collected and levels of TNF-α, IFN-γ, IL-2, IL-4, IL-5, IL-13, IL-10, IL-17A, IL-17F, IL-21, and IL-22 were assessed cells by multiplex bead assay (ProcartaPlex Mix&Match Human plex; eBioscience).

### Assessment of serum cytokine profile.

Serum levels of IL-1α, IL-6, IL-23, IL-23, and IP-10 were assessed by Luminex assay (ProcartaPlex Mix&Match Human plex; eBioscience), and CRP was assessed by nephelemetry (CardioPhasehsCRP; Siemens Healthcare Diagnostics Products GmbH) as previously described (27).

### Flow cytometry analyses.

Cells were fixed with CellFix (BD), acquired on an LSRII SORP (4 lasers: 405, 488, 532, and 633 nm), and analyzed using FlowJo (version 9.7.7) (Tree Star Inc, Ashland, OR). Frequencies of cytokine-producing M. tuberculosis-specific T cells and cytokine profiles of M. tuberculosis-specific T-cell responses were analyzed using SPICE software (version 5.34) following background subtraction. When required, analysis and presentation of distributions was performed using SPICE, downloaded from http://exon.niaid.nih.gov/spice (54).

### Statistical analyses.

Statistical significance (*P* values) was determined either using two-tailed chi-square analysis for comparison of positive proportions or using one-way analysis of variance (ANOVA; Kruskal-Wallis test) followed by Mann-Whitney test or Wilcoxon matched-pairs two-tailed signed-rank test for multiple comparisons or Spearman rank test for correlations using GraphPad Prism version 7 (San Diego, CA). Statistical analyses of global cytokine profiles (pie charts) were performed by partial permutation tests using the SPICE software as described previously (54).
